# Comparison of outpatient medically attended and community‐level influenza‐like illness—New York City, 2013‐2015

**DOI:** 10.1111/irv.12540

**Published:** 2018-02-14

**Authors:** Kate E. Russell, Ashley Fowlkes, Melissa S. Stockwell, Celibell Y. Vargas, Lisa Saiman, Elaine L. Larson, Philip LaRussa, Steve Di Lonardo, Michael Popowich, Kirsten St. George, Andrea Steffens, Carrie Reed

**Affiliations:** ^1^ Epidemic Intelligence Service Centers for Disease Control and Prevention Atlanta GA USA; ^2^ Influenza Division National Center for Immunization and Respiratory Diseases Centers for Disease Control and Prevention Atlanta GA USA; ^3^ Columbia University Medical Center New York NY USA; ^4^ NewYork‐Presbyterian Hospital New York NY USA; ^5^ New York City Department of Health and Mental Hygiene New York NY USA; ^6^ Wadsworth Center New York State Department of Health Albany NY USA

**Keywords:** community surveillance, influenza, influenza‐like illness, respiratory viruses

## Abstract

**Background:**

Surveillance of influenza‐like illness (ILI) in the United States is primarily conducted through medical settings despite a significant burden of non‐medically attended ILI.

**Objectives:**

To assess consistency between surveillance for respiratory viruses in outpatient and community settings using ILI surveillance from the Centers for Disease Control and Prevention Influenza Incidence Surveillance Project (IISP) and the Mobile Surveillance for Acute Respiratory Infections (ARI) and Influenza‐Like Illness in the Community (MoSAIC) Study.

**Methods:**

The Influenza Incidence Surveillance Project conducts ILI surveillance in 3 primary care clinics in New York City, and MoSAIC conducts community‐based ILI/ARI surveillance through text messaging among a cohort of New York City residents. Both systems obtain respiratory specimens from participants with ILI/ARI and test for multiple pathogens. We conducted a retrospective review of ILI cases in IISP and MoSAIC from January 2013 to May 2015 with descriptive analyses of clinical and laboratory data.

**Results:**

Five‐hundred twelve MoSAIC and 669 IISP participants met an ILI criteria (fever with cough or sore throat) and were included. Forty percent of MoSAIC participants sought care; the majority primary care. Pathogens were detected in 63% of MoSAIC and 70% of IISP cases. The relative distribution of influenza and other respiratory viruses detected was similar; however, there were statistically significant differences in the frequency that were not explained by care seeking.

**Conclusions:**

Outpatient and community‐based surveillance in the one found similar timing and relative distribution of respiratory viruses, but community surveillance in a single neighborhood may not fully capture the variations in ILI etiology that occur more broadly.

## 1 INTRODUCTION

Surveillance for influenza‐like illness (ILI) activity in the United States traditionally relies on reports of medically attended visits, including outpatient, and emergency room visits and hospitalizations.^1^ The majority of ILI is mild and self‐limited, and many patients never seek care in a medical setting.^2^ Surveillance for medically attended ILI misses cases of non‐medically attended ILI in the community and underestimates the true prevalence of ILI in the population. Understanding community ILI can shed light on the full burden of influenza. Furthermore, primary care clinics, a common setting for outpatient ILI surveillance, may not adequately capture cases who could seek care in other settings, such as emergency departments or urgent care clinics. In addition, systems that focus on medically attended ILI may have other biases (eg, access to care) and delays associated with care seeking that may affect sensitivity of testing and may not reflect influenza and other respiratory virus activity in the broader community.^3^, ^4^, ^5^ A comparison between community surveillance and outpatient medically attended ILI surveillance systems could help to determine how well medically attended surveillance systems reflect respiratory viral activity in the community.

Community surveillance for ILI in the United States has been performed periodically in several cohort studies in various small geographic areas^6^, ^7^, ^8^, ^9^; broader scale community surveillance is generally cost prohibitive. The Mobile Surveillance for Acute Respiratory Infections and Influenza‐Like Illness in the Community (MoSAIC) Study is one recent prospective household‐based cohort study established in 2012, in a neighborhood in New York City to obtain community‐level incidence of influenza and other respiratory viruses.^8^

In 2009, New York City, along with other participating sites, began the Influenza Incidence Surveillance Project (IISP). IISP is designed to determine the incidence of medically attended ILI and the proportion of ILI attributable to influenza and other respiratory diseases using provider estimates of patient populations.^10^

Using these two systems in the single geographic region of New York City, we sought to compare symptoms as well as the frequency, proportion, and seasonal distribution of respiratory pathogens associated with ILI between individuals with medically attended ILI and broader community‐level ILI across three influenza seasons to better understand how current ILI surveillance in medical clinics reflects activity seen at the community level.

## 2 METHODS

### 2.1 MoSAIC surveillance

The MoSAIC study methods have been described previously.^8^ Briefly, MoSAIC is a CDC‐funded community‐based study which performs surveillance for acute respiratory illness (ARI), including influenza‐like illness (ILI), in a neighborhood in New York City year‐round with the goal to assess ARI and ILI rates and etiology in the community. The cohort consists of approximately 250 households and is a primarily Latino population. Demographic information is collected upon enrollment. Households receive twice‐weekly text messages, asking if anyone has runny nose, congestion, sore throat, cough, body aches, or fever and report either “yes” or “no.” Home visits are performed by research staff to obtain a nasal swab from any participants meeting the ARI criteria (2 symptoms including fever, runny nose/congestion, sore throat, cough, and/or myalgia) with symptoms lasting < 5 days and who are still symptomatic. The ARI criteria for infants (less than 12 months of age) also include runny nose/congestion alone. Respiratory swabs are analyzed in a research laboratory using a BioFire FilmArray multiplex polymerase chain reaction (PCR) assay (BioFire Diagnostics LLC, Salt Lake City, UT) detecting influenza A virus (H1, H1N1pdm09, and H3), influenza B virus, adenovirus, coronaviruses (229E, HKU1, NL63, and OC43), enteroviruses/rhinoviruses, human metapneumovirus (HMPV), human parainfluenza viruses (HPIV) types 1‐4, respiratory syncytial virus (RSV), *Chlamydia pneumoniae*,* Mycoplasma pneumoniae*, and *Bordetella pertussis*. Further information on symptoms and care‐seeking behaviors is collected by interview at illness end. We limited our analysis to episodes meeting ILI criteria (fever with cough or sore throat) for appropriate comparison to ILI within IISP.

### 2.2 IISP surveillance

The CDC's IISP is a population‐based outpatient surveillance network operating year‐round, for syndromic ILI with systematic laboratory testing for influenza viruses, including three primary care clinics in New York City.^10^ Since 2009, participating clinics report the weekly number of ILI and all‐cause visits. Patients with symptom onset within 7 days of presentation are included, and ILI is defined as fever with cough or sore throat among patients ≥ 2 years of age, and fever with ≥1 of cough, sore throat, nasal congestion, or rhinorrhea among children < 2 years of age. Nasopharyngeal (NP) or oropharyngeal (OP) swabs are collected from the first 10 ILI patients of each week and tested at the NYC Public Health Laboratory for influenza using the CDC real‐time RT‐PCR assay. Specimens collected from October 2011 through August 2014 were further tested for respiratory pathogens by the New York State Department of Health Wadsworth Center using Taqman Array Card^11^, ^12^ (TAC, Life Technologies, Carlsbad, CA) that included influenza A virus (H1, H1N1pdm09, and H3), influenza B virus, adenovirus, coronaviruses (229E, HKu1, NL63, OC43), enterovirus, HMPV, HPIV types 1‐4, RSV, rhinovirus, *Chlamydia pneumoniae*,* Mycoplasma pneumoniae*,* Bordetella pertussis*,* Haemophilus influenzae, Legionella pneumophila*,* Streptococcus pneumoniae*,* Streptococcus pyogenes*. Discordant results in influenza virus testing between the CDC PCR testing kits and Taqman testing were assumed positive by any assay. Specimens collected from August 2014 through May 2015 were also tested using the Luminex xTAG^®^ Respiratory Virus Panel (RVP; Luminex Diagnostics, Toronto, Canada) for RSV, HPIV types 1‐4, HMPV, rhino/enteroviruses (non‐distinguishing target), and adenovirus.

### 2.3 Statistical analysis

A retrospective analysis of MoSAIC and IISP and surveillance data collected from January 2013 to May 2015 was performed. Included cases were those in persons with symptom onset between morbidity and mortality weekly report (MMWR) weeks 40 through 20 (approximately early October through mid‐May) of each influenza season. We limited comparisons to viral pathogens common to the Taqman, Biofire, and Luminex assays, specifically influenza A virus, influenza B virus, adenovirus, coronaviruses, enteroviruses/rhinoviruses, HMPV, HPIV types 1‐4, and RSV. Descriptive analyses were conducted on ILI episodes reported from the MoSAIC and NYC IISP site populations for demographic characteristics of age, gender, and whether participants had received the seasonal influenza vaccine at least 14 days prior to illness onset. Episodes in a single individual that were at least 14 days apart were included as separate illness episodes. Symptoms reported in MoSAIC and IISP populations were compared using a set of log‐binomial regression models adjusted for age to estimate the relative risk of individual symptoms among persons with ILI reported through community‐level surveillance compared with medical provider surveillance. A second set of age‐adjusted log‐binomial regression models was used to estimate the relative prevalence of individual pathogens detected in persons with ILI through MoSAIC compared to IISP. Due to incomplete data, vaccination was not adjusted for. A subset analysis was performed comparing MoSAIC participants who sought care to IISP participants. Correlation coefficients were calculated for seasonal distribution of influenza and RSV viruses between the two surveillance systems. Statistical significance was set at .05, and analyses were performed using SAS (v 9.3) software.

## 3 RESULTS

### 3.1 Study populations and symptoms

During the surveillance period, 334 households were followed by MoSAIC with an estimated 4.8 persons per household. The estimated population under surveillance among the three IISP sites was 20 368. A total of 512 MoSAIC and 669 IISP participants meeting the ILI case definition over the study period were tested by RT‐PCR (Table 1). The ILI cases identified through IISP had a slightly higher proportion of adults compared with MoSAIC cases, while the MoSAIC cases had a higher proportion of females. Forty percent of MoSAIC participants with ILI sought care for their illness. Of those who did seek care, most (81%) sought care in a primary care setting, similar to clinics represented in IISP, while the remaining cases were seen in an emergency department, urgent care clinic, or retail clinic (Table 1). Influenza vaccination data were reported for 351 (69%) of MoSAIC participants and 421 (63%) of IISP participants. Of these, 160 (46%) of the MoSAIC participants and 118 (28%) of IISP participants had received a seasonal influenza vaccine at least 14 days prior to the reported illness episode. ILI was associated with referral for hospitalization for one (0.2%) participant in the MoSAIC group and 2 (0.8%) participants in the IISP group. Median time from symptom onset to date of testing was 2 days for both MoSAIC and IISP.

**Table 1:** Demographics and clinical characteristics of the New York City Influenza Incidence Surveillance Project (IISP) and Mobile Surveillance for Acute Respiratory Infections and Influenza‐Like Illness in the Community (MoSAIC) study populations enrolled during 3 influenza seasons from January 2013 to May 2015 with Influenza‐like illness

	IISP, n = 669n (%)	MoSAIC, n = 512n (%)
Age		
<5 y	148 (22)	138 (27)
5‐17 y	124 (19)	168 (33)
18‐49 y	250 (37)	140 (27)
≥50 y	147 (22)	66 (13)
Gender		
Female	355 (53)	324 (63)
Male	313 (47)	188 (37)
Received seasonal flu vaccine	118/421 (28)	160/351 (45)
Care seeking		
None	0 (0)	309 (60)
Primary care	669 (100)	167 (33)
Retail clinic	0 (0)	2 (0.4)
Urgent care	0 (0)	6 (1)
ED	0 (0)	31 (6)
Hospitalized	2 (1)	1 (0.2)
Days from symptom onset to specimen collection		
Median (IQR)	2 (1,3)	2 (1,4)

While all participants included in this analysis met the case definition of ILI (fever and cough or sore throat), there were significant differences in symptoms reported by participants in MoSAIC as compared with participants in IISP (Table 2). Differences persisted even after adjusting for age; ILI cases in MoSAIC were more likely to report rhinorrhea (81% vs 62%, *P* < .01) and less likely to report sore throat (47% vs 61%, *P* < .01) or myalgia (23% vs 46%, *P* < .01) compared with those in IISP. To further explore if these difference were due to care‐seeking behavior, we examined reported symptoms among only MoSAIC cases that reported seeking care for their illness and found that differences compared with the IISP group persisted (Table 2).

**Table 2:** Symptoms of ILI cases among the New York City Influenza Incidence Surveillance Project (IISP) population, Mobile Surveillance for Acute Respiratory Infections and Influenza‐Like Illness in the Community (MoSAIC) population, and participants of MoSAIC seeking care for ILI, adjusted for age

	IISP^a^, (n = 669)	MoSAIC—all, (n = 512)			MoSAIC—sought care, (n = 203)		
	n (%)	n (%)	aRR	CI	n (%)	aRR	CI
Fever	669 (100)	512 (100)	‐	‐	203 (100)	‐	‐
Cough	528 (79)	423 (83)	1.03	0.98‐1.09	175 (86)	1.06	1.00‐1.12
Sore throat	411 (61)	241 (47)	0.86	0.78‐0.95^b^	84 (41)	0.82	0.69‐0.97^b^
Rhinorrhea	412 (62)	413 (81)	1.32	1.22‐1.42^b^	164 (81)	1.32	1.20‐1.44^b^
Myalgia	305 (46)	119 (23)	0.67	0.57‐0.79^b^	33 (16)	0.62	0.45‐0.85^b^

aRR, age‐adjusted risk ratio.

a Reference group.

b Indicates statistical significance with *P* < .05.

### 3.2 Comparison of pathogen detections among MoSAIC and IISP

**Figure 1:**
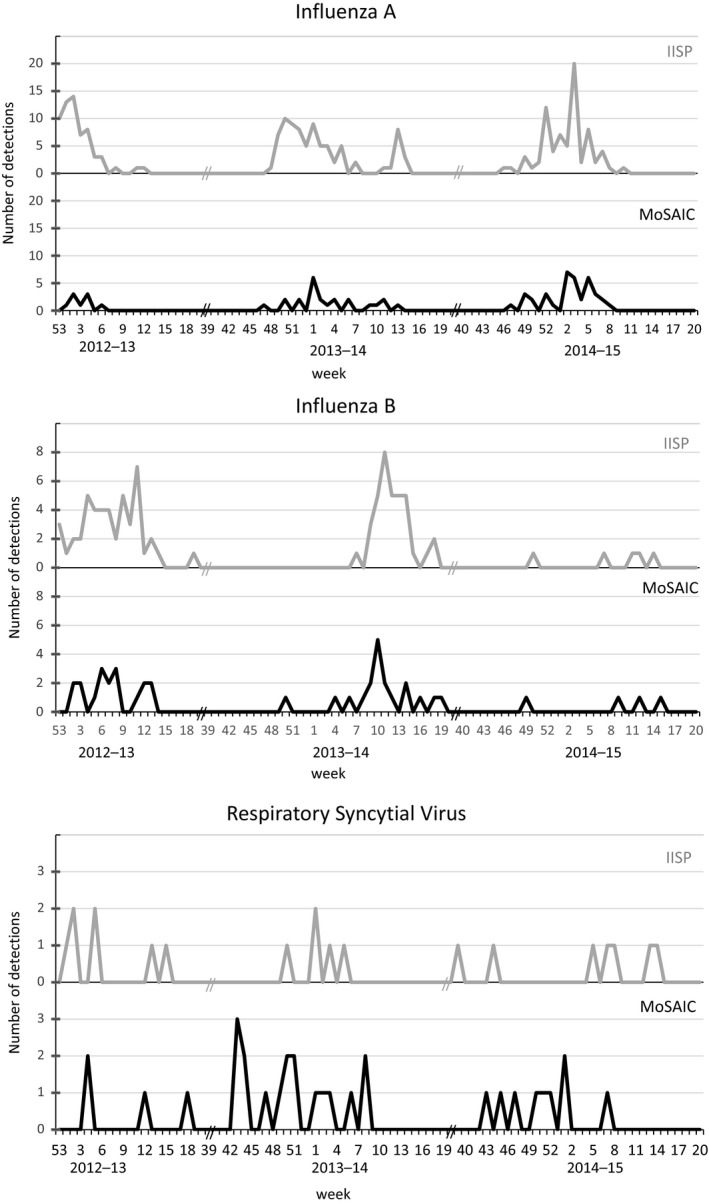
Seasonal distribution of influenza A viruses, influenza B viruses, and respiratory syncytial viruses (RSV) by week in the New York City Influenza Incidence Surveillance Project (IISP) and the Mobile Surveillance for Acute Respiratory Infections and Influenza‐Like Illness in the Community (MoSAIC) Study among episodes of influenza‐like illness

Overall, 324 (63%) of 512 MoSAIC ILI cases and 468 (70%) of 669 IISP cases were positive for at least one of the pathogens tested and included in the analysis. The seasonal distribution of influenza A and influenza B viruses by week were similar between MoSAIC and IISP with correlation coefficients of .54 and .61, respectively (Figure 1). Distribution of peaks of RSV detection and seasonality was less well defined in the two groups with a correlation coefficient of −.01 (Figure 1).

Influenza A virus and rhinovirus/enterovirus were the most frequently detected pathogens in both MoSAIC and IISP, although percentages varied (Table 3). Influenza A and B viruses were less frequently detected in MoSAIC compared with IISP (14% and 8% in MoSAIC; 34% and 14% in IISP, adjusted risk ratio (aRR) 0.4 and 0.5, respectively). HMPV and RSV detections were more frequently detected in MoSAIC than IISP (5.3% and 5.9% in MoSAIC; 3.1% and 3.3% in IISP, aRR 1.8 and 1.8, respectively).

**Table 3:** Proportion of pathogens detected among the New York City Influenza Incidence Surveillance Project (IISP) population, Mobile Surveillance for Acute Respiratory Infections and Influenza‐Like Illness in the Community (MoSAIC) population with influenza‐like illness (ILI), and participants of MoSAIC seeking care for ILI, during 3 influenza seasons from January 2013 to May 2015

	IISP^a^, (n = 669)	MoSAIC—all, (n = 512)			MoSAIC—sought care, (n = 203)		
	n (%)	n (%)	aRR	CI	n (%)	aRR	CI
Adenovirus	19 (3)	6 (1)	0.34	0.14‐0.83^c^	5 (2)	0.54	0.20‐1.43
Coronavirus	25 (7)	47 (9)	1.35	0.83‐2.19	11 (5)	0.94	0.45‐1.98
Human metapneumovirus	19 (3)	27 (5)	1.80	1.01‐3.21^c^	17 (8)	2.60	1.34‐5.05^c^
Influenza A virus	229 (34)	69 (14)	0.40	0.31‐0.51^c^	26 (13)	0.37	0.26‐0.55^c^
Influenza B virus	91 (14)	41 (8)	0.55	0.38‐0.78^c^	18 (9)	0.60	0.37‐0.98^c^
Parainfluenza virus	10 (2)	8 (2)	1.07	0.42‐2.72	3 (1)	0.99	0.28‐3.81
Respiratory syncytial virus	20 (3)	30 (6)	1.85	1.05‐3.24^c^	16 (8)	2.23	1.14‐4.35^c^
Rhinovirus/Enterovirus	102 (17)	110 (21)	1.15	0.91‐1.47	49 (24)	1.20	0.88‐1.64
Co‐detection	47 (7)	16 (3)	0.41	0.24‐0.71^c^	9 (4)	0.51	0.25‐1.02
Negative^b^	201 (30)	188 (37)	1.29	1.10‐1.52^c^	65 (32)	1.21	0.95‐1.55

aRR, age‐adjusted risk ratio.

a Reference group.

b Negative for the above‐mentioned pathogens.

c Indicates statistical significance with *P* < .05.

To examine if these differences in proportions were due to care seeking, we examined detection of viral pathogens among only MoSAIC participants who reported seeking care for ILI with IISP data and observed similar differences in the trends of viral detections (Table 3). Influenza A and B viruses were still more frequently detected in IISP (13% and 9% among care seeking in MoSAIC participants; 34% and 14% in IISP participants, aRR 0.4 and 0.6, respectively). HMPV and RSV remained more frequent among care‐seeking MoSAIC participants compared with IISP (8.4% and 7.9% among care seeking in MoSAIC; 3.1% and 3.3% in IISP, aRR 2.6 and 2.2, respectively).

Differences were also seen by age group (Table 4). Detection of any virus among older adults (≥50 years) was higher in IISP. Among children < 5 years of age, influenza A and B were detected more frequently in IISP compared with MoSAIC (35% vs 4.3%, and 7% vs 4%, respectively), whereas RSV was detected more frequently in the MoSAIC population (12% vs 4%, respectively). Similarly, among children 5‐17 years of age, influenza A and B were detected more frequently in IISP compared with MoSAIC (34% vs 19%, and 26% vs 10%, respectively), although RSV was detected was similar. The frequency of detection for most viruses was more similar between the MoSAIC and IISP populations as age increased. The frequency of detection for most viruses was more similar between the MoSAIC and IISP as age increased. Influenza A remained higher in IISP throughout.

**Table 4:** Frequency of viruses detected by age group among the New York City Influenza Incidence Surveillance Project (IISP) and Mobile Surveillance for Acute Respiratory Infections and Influenza‐Like Illness in the Community (MoSAIC) populations with influenza‐like illness during 3 influenza seasons from January 2013 to May 2015

	<5 y		5‐17 y		18‐49 y		≥50 y	
	IISP, n = 148	MoSAIC, n = 138	IISP, n = 124	MoSAIC, n = 168	IISP, n = 250	MoSAIC, n = 140	IISP, n = 147	MoSAIC, n = 66
	n (%)	n (%)	n (%)	n (%)	n (%)	n (%)	n (%)	n (%)
Adenovirus	13 (9)	5 (4)	2 (2)	0 (0)	2 (1)	1 (1)	2 (1)	0 (0)
Coronavirus	7 (5)	10 (7)	1 (1)	16 (10)	11 (4)	14 (10)	6 (4)	7 (11)
Human metapneumovirus	6 (4)	19 (14)	1 (1)	3 (2)	4 (2)	2 (1)	8 (5)	3 (5)
Influenza A	52 (35)	6 (4)	42 (34)	32 (19)	78 (31)	18 (13)	57 (39)	13 (20)
Influenza B	11 (7)	5 (4)	32 (26)	16 (10)	28 (11)	11 (8)	20 (14)	9 (14)
Parainfluenza virus	3 (2)	4 (3)	1 (1)	0 (0)	5 (2)	4 (3)	1 (1)	0 (0)
Respiratory syncytial virus	6 (4)	17 (12)	2 (2)	5 (3)	6 (2)	6 (4)	6 (4)	2 (3)
Rhinovirus/Enterovirus	36 (24)	47 (34)	15 (12)	40 (24)	43 (17)	19 (14)	8 (5)	4 (6)

## 4 DISCUSSION

Using data from two separate surveillance systems that incorporate molecular testing for multiple respiratory viruses in the same city, we compared the frequency and seasonal distribution of circulating viruses among individuals with ILI in the community and patients attending outpatient clinics. Only a third of the community MoSAIC participants sought care for ILI in a primary care clinic, indicating that surveillance in this setting would miss the majority of the burden of ILI in the community. This finding has been observed in previous telephone‐based surveys of individuals in the community with ILI which found a similar proportion of those with ILI reported that they sought medical care as we found in the MoSAIC group.^4^ Community surveillance systems, such as MoSAIC, are necessary to capture the full burden of influenza and other causes of ILI across levels of disease severity and are not biased by factors that influence care seeking. Among medically attended illnesses, most MoSAIC participants with ILI did seek care at a primary care location as opposed to urgent care, retail clinics or emergency departments, thus the IISP surveillance system represents the majority of medically attended community illnesses by sampling from primary care clinics including pediatric, internal medicine, and family practices clinics.^10^

The general pattern of proportion of viruses detected among ILI episodes; the two surveillance systems were similar; the most common viruses detected in both systems were rhinovirus/enteroviruses, RSV and influenza viruses. The most infrequently detected viruses in both systems were HPIVs and adenovirus. Both systems had similar seasonal distributions of influenza A and B viruses, indicating that the two systems capture the timing of influenza activity equally well. A major advantage of community‐level surveillance is the potential for more rapid detection of changes in circulating virus given delays associated with care seeking that have been seen previously.^4^ However, this was not seen in the comparison of these two systems. Testing results in MoSAIC are known to the surveillance system in near real‐time, whereas medically attended ILI surveillance is usually summarized for the week with at least a week delay. Although samples were collected at similar time points after symptom onset in both MoSAIC and IISP, other medically attended surveillance systems have noted delays in testing associated with care seeking.^3^

Despite the similar general patterns observed among ILI in both surveillance systems, there were statistically significant differences in the frequency of detection of most viruses that were not completely explained by care seeking. Significant differences were also observed when comparing only the medically attended illnesses in MoSAIC with the IISP population. There may be several possible reasons for this finding including differences in the surveillance system methods, differences in the population demographics, household clustering, or potentially limited geographic variation in the circulation of the viruses detected. The two surveillance populations differ in that the MoSAIC population is mostly publicly insured and Latino and, while IISP does not collect demographic information on participants, they are primarily private practices and may be more likely to have commercially insured patients. Care seeking is both less frequent and less timely among persons without insurance and also varies with demographics such as age, or education.^4^, ^8^, ^13^ Clinics may have a higher proportion of persons with underlying illnesses as compared with the general community. Also, the three contributing clinics in IISP likely have patients from several neighborhoods across the city while MoSAIC recruits from a single neighborhood. These two factors may contribute to differences in social mixing within each of the two study groups, despite residing in the same city. While state‐to‐state variation in influenza activity is seen in the United States in national surveillance data, there have been limited studies on small‐scale geographic variations in respiratory pathogen circulation. However, local variation in RSV epidemics and lower respiratory illness clustering have been described previously.^14^, ^15^, ^16^

A possible disadvantage of community surveillance is that the intensive cohort study used in MoSAIC with in‐person specimen collection and monthly home visits would likely be cost prohibitive on a broader scale. Further efforts to reduce costs could include relying on automated text messaging or email with follow‐up and self‐swabbing, which may allow community surveillance to be used more broadly.^17^, ^18^

### 4.1 Limitations

Our analysis had several limitations. Firstly, we restricted to an ILI definition to focus on influenza, however, may not be optimal for the other viral pathogens tested. Our objective was to compare the pathogens detected within the context of ILI between the two systems rather than the full burden of all pathogens. Data on vaccination were incomplete in both IISP and MoSAIC, limiting our ability to control for vaccination in the analysis. In addition, we did not take possible household clustering into account among MoSAIC participants who were living in the same residence.

## 5 CONCLUSIONS

Surveillance through community cohorts can more fully capture local incidence and etiology of ILI and has the potential to provide further information beyond what is capable in medically attended surveillance, such as transmissibility^8^, ^19^, ^20^, ^21^ and may add to our understanding of the full burden of influenza. While the timing of influenza and other virus detections were captured equally well in community and outpatient surveillance, the proportions of viruses detected varied between the community and outpatient clinics. There may be local geographic and/or social mixing differences affecting incidence of circulating viruses at a very local level and outpatient surveillance across more clinics may better reflect viral circulation in the larger community. Community and medically attended surveillance are valuable and complementary for understanding full ILI incidence, etiology, and burden.

## DISCLAIMER

The contents of this article are solely the responsibility of the authors and do not necessarily represent the official view of the Association of Public Health Laboratories or the Centers for Disease Control and Prevention.
